# Polarizing receptor activation dissociates fibroblast growth factor 2 mediated inhibition of myelination from its neuroprotective potential

**DOI:** 10.1186/s40478-019-0864-6

**Published:** 2019-12-19

**Authors:** Katja Thümmler, Eran Rom, Thomas Zeis, Maren Lindner, Sarah Brunner, John J. Cole, Diana Arseni, Steve Mücklisch, Julia M. Edgar, Nicole Schaeren-Wiemers, Avner Yayon, Christopher Linington

**Affiliations:** 1 Institute of Infection, Immunity and Inflammation, University of Glasgow, 120 University Place, Glasgow, G12 8TA UK; 2 ProCore Bio Med Ltd., Weizmann Science Park, 70400 Ness Ziona, Israel; 3 Neurobiology Laboratory, University Hospital Basel, University of Basel, 4031 Basel, Switzerland; 4 Department of Computer Science, Chemnitz University of Technology, 09111 Chemnitz, Germany

**Keywords:** Multiple sclerosis and Neuroinflammation, Remyelination, Neuroprotection, Neuroinflammation, Oligodendrocyte

## Abstract

Fibroblast growth factor (FGF) signaling contributes to failure of remyelination in multiple sclerosis, but targeting this therapeutically is complicated by its functional pleiotropy. We now identify FGF2 as a factor up-regulated by astrocytes in active inflammatory lesions that disrupts myelination via FGF receptor 2 (FGFR2) mediated activation of Wingless (Wnt) signaling; pharmacological inhibition of Wnt being sufficient to abrogate inhibition of myelination by FGF2 in tissue culture. Using a novel FGFR1-selective agonist (F2 V2) generated by deleting the N-terminal 26 amino acids of FGF2 we demonstrate polarizing signal transduction to favor FGFR1 abrogates FGF mediated inhibition of myelination but retains its ability to induce expression of pro-myelinating and immunomodulatory factors that include *Cd93*, *Lif*, *Il11*, *Hbegf*, *Cxcl1* and *Timp1*. Our data provide new insights into the mechanistic basis of remyelination failure in MS and identify selective activation of FGFR1 as a novel strategy to induce a neuroprotective signaling environment in multiple sclerosis and other neurological diseases.

## Introduction

Multiple sclerosis (MS) is a chronic neurodegenerative disease of the central nervous system (CNS) in which repeated episodes of inflammatory demyelination result in persistently demyelinated plaques of gliotic scar tissue associated with varying degrees of axonal injury and loss. This axonal pathology is the underlying cause of chronic disability in MS and is intimately associated with demyelination. Loss of myelin in the inflammatory milieu of an MS lesion not only increases axonal susceptibility to inflammatory mediators per se [63] but also disrupts metabolic support provided by myelinating oligodendrocytes [28, 50]; a combination of effects that result in profound axonal energy deficits that compromise the functional and structural integrity of affected axons [12, 26, 81]. In experimental models these detrimental effects of demyelination on axonal health are mitigated by remyelination carried out by oligodendrocytes derived from oligodendrocyte progenitor cells (OPC) [21, 43, 52, 93]. However, in MS this endogenous repair mechanism frequently fails leaving demyelinated axons increasingly vulnerable to inflammatory and metabolic stress [27].

Why remyelination fails in MS remains unclear, but it is generally believed to involve factors that disrupt the differentiation of OPC into myelination-competent oligodendrocytes [15, 48, 73, 84]. These include changes affecting growth factor availability and composition of the extracellular matrix, inhibitory signals derived from myelin debris, inappropriate re-expression of developmental signaling pathways, and effects due to ageing [74]. However, the initial events that disrupt OPC differentiation in the inflammatory milieu of an MS lesion remain obscure. Recent studies suggest this may involve members of the fibroblast growth factor (FGF) family, in particular FGF2 [18, 39, 54, 69] which acts as a negative regulator of (re) myelination in the adult CNS [10, 53].

FGF2 plays important roles in CNS development during which it influences proliferation, speciation and migration of neural progenitor cells [85]. It continues to be expressed at low levels by astrocytes and some neurons in the adult CNS, but is rapidly up regulated in response to CNS injury [58]. The pathophysiological significance of this response is unclear as FGF2 is functionally pleiotropic, experimental studies demonstrating that in addition to its detrimental effects on remyelination it also supports OPC proliferation and migration [2, 9, 11], as well as mediating a neuroprotective response which reduces clinical deficits and tissue damage in animal models of MS [66, 68]. Any attempt to modulate FGF2 signaling to enhance lesion repair must take this functional pleiotropy into account and aim to suppress its detrimental effects on remyelination whilst retaining its “neuroprotective” potential.

Canonical members of the FGF family such as FGF2 signal via a small family of transmembrane tyrosine kinases encoded by *Fgfr1*, *Fgfr2*, *Fgfr3* and *Fgfr4* which activate a variety of intracellular signaling pathways including RAS-MAPK, PI3K-AKT, PLCγ, and signal transducer and activator of transcription (STAT) (reviewed in [57]). Previous studies demonstrate the biological outcome of FGF2 signaling within the oligodendrocyte lineage is determined by stage-specific changes in receptor expression; activation of FGFR1 driving OPC proliferation, whilst subsequent and sequential expression of FGFR3 and FGFR2 on oligodendrocytes is associated with inhibition of myelin protein expression and de-differentiation [25]. But does this concept extend to the complex environment of the CNS in which these receptors are also expressed by astrocytes, glia, neurons, microglia and endothelial cells? Specifically, would skewing signal transduction to favour FGFR1 suppress its detrimental effects of myelination whilst retaining its ability to support OPC proliferation and generate a broadly „neuroprotective “signaling environment.

We report expression of FGF2 by astrocytes correlates with inflammatory activity in MS lesions and present data demonstrating this inhibits myelination via FGFR2-mediated activation of Wingless (Wnt)-signaling; pharmacological inhibition of Wnt signal transduction being sufficient to abrogate the inhibition of myelination by FGF2 in tissue culture. Skewing signal transduction to favour FGFR1 abolishes this detrimental effect on OPC differentiation, but retains the ability of FGF2 to act as an OPC mitogen and induce expression of “neuroprotective” factors with anti-inflammatory, neuroprotective and pro-myelinating properties. Our data demonstrate the biological outcome of FGF2 signaling in the CNS is determined at the level of FGFR usage and raises the exciting possibility FGFR1-specific agonists may provide a new approach to enhance lesion repair in the CNS.

## Materials and methods

### Generation of F2 V2 and FGFR specificity assay

NdeI and BamHI sites were appended to human FGF2 cDNA by PCR and the resulting fragment was cloned in NdeI/BamHI digested pET9a. A deletion mutant of FGF2 lacking the N-terminal 26 amino acids of the native protein (F2 V2) was designed and generated by oligonucleotide directed PCR mutagenesis of pET9aFGF2 using the following primers as described in US patent WO2008/038287:

FGF2Δ26-F 5’GGAATTCCATATGAAGGACCCCAAGCGGCTG.

FGF2-R 5’CGGGATCCTCAGCTCTTAG.

The resulting pET9aFGF2^Δ26^ was expressed in BL21DE3 bacteria and the product (F2 V2) purified on heparin-Sepharose column (US patent WO2008/038287).

To define receptor specificity, the mouse myeloid progenitor cell line FDCP-1 was cultured in ISCOVES medium [(Gibco, Rockville, MD, USA) supplemented with 10% FCS, penicillin, streptomycin, glutamine and 0.1 ng/ml IL3] and transfected with full length human FGFR1, 2, 3 or 4 (FDCP-FGFR1, FDCP-FGFR2, FDCP-FGFR3, FDCP-FGFR4). Transfected FDCP-1 cells were plated at a density of 2 × 10^4^ cells/well in 96 well plates in the same medium, but substituting IL3 with 10 ng/ml of either FGF2 or F2 V2. Proliferation was determined 48 h later using XTT Cell Proliferation Assay (Biological Industries, Beit Haemek, Israel). The FGFR specific human scFv antibodies PRO-001 (FGFR3 specific) and PRO-007 (FGFR2/3 specific), generated using phage display libraries [62] and produced by bacterial fermentation at Fibron Ltd. Israel were used as described previously [80].

### Human tissues: in situ hybridization

In situ hybridization studies were carried out using fresh frozen tissue samples provided by the UK Multiple Sclerosis Tissue Bank (UK Multicentre Research Ethics Committee, MREC/02/2/39). Synthetic digoxigenin-labelled riboprobes (cRNA) were generated from recombinant pCRTMII-Topo® plasmid containing a 691 bp cDNA insert of human FGF2 (sequence: 5′-2985 to 3675–3′). Transcription was done from both sides with either SP6 or T7 RNA polymerase, generating antisense or sense (control) cRNA probes. In situ hybridization was performed on cryosections of freshly frozen tissues as described previously [35, 71]. In situ hybridization signals were revealed by alkaline phosphatase with BCIP (5-bromo- 4-chloro-30-indolyphosphate) and NBP (nitro-blue tetrazolium) as substrate.

### Immunohistochemistry and immunofluorescence of human tissues

Tissue sections were fixed in 4% PFA for 15 min and then washed twice with PBS. Thereafter sections were treated with 0.6% hydrogen peroxide in methanol for 30 min and with blocking buffer (1% normal donkey serum, 0.1% TritonTM X-100, 0.05% Tween) for 1 h. Sections were then incubated with the following primary antibodies overnight at 4 °C: rabbit anti-OLIG2 (1:500, Millipore), rabbit anti-GFAP (1:2000, DakoCytomation), mouse anti-MOG (1:500, Z12) and rat anti-CD68 (1:500, Abcam). Sections were washed with PBS and then secondary biotinylated antibodies (Vector Laboratories, 1:500) were applied for 2 h at room temperature, followed by ABC complex reagent (Vector Labs) for 30 min. Colour reaction was performed with 3-amino-9-ethylcarbazole. Luxol Fast blue and haematoxylin and eosin staining were performed according to standard protocols. Immunofluorescent stainings were made after blocking in 5% NDS, 1% FSG, 0.3 M glycine for 2 h. Sections were then incubated with rabbit anti-FGF2 (1:500, Abbiotec), rabbit anti-FGFR1 (1:500, Abbiotec) and/or mouse anti-GFAP (1:1000, Sternberger Monoclonals Inc.). Autofluorescence was quenched by incubating the slides 1 h in 10 mM CuSO_4_ in 50 mM Ammonium acetate buffer (pH 5.0). Sections were washed in PBS and incubated with secondary antibodies (1:500, Jackson Immunoresearch Europe Ltd.) and 1:10000 DAPI for 2 h at RT. Sections were then washed with PBS and mounted with Fluorosave (MerckMillipore). For each data point the average total intensity of 10 randomly taken pictures was measured, log2 transformed and normalized to the corresponding value of the normal appearing white matter (within the same patient).

### Cell and tissue culture

In vitro myelinating cultures were established from embryonic day 15.5 rat spinal cords (Sprague Dawley) or embryonic day 13.5 mouse spinal cords (C57BL/6 J) as described previously [22, 51, 78]. Cultures were maintained at 37 °C/7% CO_2_ and fed three times a week by replacing half the culture medium with fresh differentiation media. Twelve days later insulin was omitted from the culture medium to promote myelination. Cultures were treated with the following factors as detailed in the text: recombinant human F2 V2 (10, 50 or 100 ng/ml), FGFR3 blocking antibody PRO-001 (10 μg/ml) and FGFR2&3 blocking antibody PRO-007 (5 μg/ml) (all provided by ProCore Biomed. Ltd. Israel); human FGF2 (10, 50 or 100 ng/ml, Peprotech); 20 μM Tankyrase Inhibitor XAV939 (Tocris).

Neurosphere-derived astrocytes were generated as described previously [51] by dissociating striata of post-natal day 1 Sprague-Dawley rats or C57BL/6 J mice and resuspending them in 20 ml neurosphere media (see [51]) supplemented with 20 ng/ml mouse submaxillary gland epidermal growth factor (EGF, R&D Systems) in a 75 cm^3^ tissue culture flask. Triturated neurospheres were then plated on PLL coated cover slips in low glucose DMEM supplemented with 10% foetal bovine serum and cultured until they formed a confluent monolayer.

OPCs were immunopurified from P1 rat cortex using an anti-A2B5 MicroBead Kit (Miltenyi Biotec, Germany) according to manufacturer’s instructions. Purified A2B5^+^ progenitors were plated on PLL coated cover slips in Basal Chemically Defined medium (DMEM, 4 mM L-glutamine, 1 mM sodium pyruvate, 0.1% BSA, 50 μg/ml Apo-transferrin, 5 μg/ml insulin, 30 nM sodium selenite, 10 nM D-Biotin and 10 nM hydrocortisone) containing 50 ng/ml PDGF and 50 ng/ml FGF2 (both Peprotech) at a density of 1–2 × 10^4^ cells / cm^2^. After 2 DIV PDGF/FGF2 was withdrawn and cells were allowed to differentiate for 6 days in modified Sato’s medium ([8]; DMEM containing 4500 mg/L glucose, 2 mM glutamine, 5000 U/ml penicillin, 5 μg/ml streptomycin, 10 μg/ml insulin, 100 μg/ml apotransferrin, 16.1 μg/ml putrescine, 60 ng/ml progesterone, 30 nM sodium selenite, 0.4 μg/ml triiodo-L-thyronine, 0.4 μg/ml L-thyroxine T4, and 0.1 mg/ml BSA) in the absence or presence of FGF2 (100 ng/ml) or F2 V2 (100 ng/ml). Cells were fed twice a week by replacing half the culture supernatant with fresh media.

### Immunofluorescence microscopy

Cultures were fixed in 4% paraformaldehyde (PFA) for 20 min at RT, permeablised with 0.5% Triton-X in PBS for 15 min and blocked for 30 min in PBS/10% Horse serum/1% bovine serum albumin. Primary antibodies were applied for 45 min and washed in PBS before secondary antibodies were applied (15 min) in the dark. Thereafter cover slips were washed with PBS followed by distilled water and mounted with Mowiol 4–88 (Calbiochem, UK). The following primary antibodies were used: SMI-31 (1:1500, mouse IgG1, Abcam), Z2 (1:500, MOG-specific, mouse IgG2a, [60]), O4 (1:500, mouse IgM, R&D Systems), Olig2 (1:1000, rabbit IgG, Millipore), AA3 (1:100, PLP/DM20 specific, rat IgG, [87]). Species and isotype specific secondary antibodies labelled with Alexa Fluor 488 or Alexa Fluor 568 (Invitrogen) were used at 1:400. OPC cell proliferation was analyzed using the Click-iT EdU Alexa Fluor 594 Kit (Invitrogen) following the manufacturer’s instructions after incubation with EdU for 72 h. EdU labelled cultures were then co-stained with DAPI and lineage-specific markers. For quantitative analysis 10 random images from each of three coverslips were taken at 10× magnification (neurite density and myelination) or 20x magnification (cell counts) using an Olympus BX51 fluorescent microscope and Image-Pro (Media Cybernetics) or Ocular software (QImaging). Representative confocal images were obtained at 63x magnification using a Zeiss LSM 710 inverted confocal microscope and Zen Black software. Neurite density, myelination and cell counts were quantified using CellProfiler cell image analysis software [14]. The pipelines developed for this study are available at https://github.com/muecs/cp.

### RNA extraction and microarray analysis

RNA was extracted using the Qiagen RNeasy Micro kit according to manufacturer’s instructions. RNA quality and integrity was checked using the Agilent Bioanalyzer 6000 Nano LabChip platform. RNA was then used for microarray expression analysis and quantitative reverse transcription (qRT)-PCR. The total RNAs were processed and labelled with biotin using Ambion® WT Expression Kit following the Affymetrix GeneChip® WT Terminal Labeling and Hybridization protocol. The processed RNAs were hybridized to Affymetrix GeneChip® Rat Gene 2.1 ST Arrays using manufacturer’s protocols for using the Fluidics Station 450. The hybridized arrays were scanned on the Affymetrix GeneTitan Scanner. Data analysis was carried out in Partek Genomics Suite (version 6.6, Partek Inc., St. Louis, MO, USA) software. Control (CTR) and Treatment (FGF) groups were generated with four replicates per group.

This dataset has been deposited in the Gene Expression Omnibus database: https://www.ncbi.nlm.nih.gov/geo/query/acc.cgi?acc=GSE65466.

The probe set level data were normalized using GCRMA normalization method and summarized to transcript cluster level using one-Step Tukey’s Biweight method. Differential expression analysis was carried out by performing one-way ANOVA test on the normalized expression values. Differentially expressed gene lists were generated based on the ANOVA with fold change > ± 1.4 at a FDR adjusted *p*-value of < 0.05 and analysed in Partek Pathway for enriched pathways utilizing the KEGG (Kyoto Encyclopedia of Genes and Genomes) database for rat. Hierarchical cluster analysis of differentially expressed genes (FDR adjusted *p*-value of < 0.05), differential expression profile analysis and gene ontology enrichment was performed with Searchlight2 [19]. Using the array background, three differential expression workflows (control vs F2 V2, control vs FGF2 and F2 V2 vs FGF2), and one multiple differential expression workflow (combining all three differential expression comparisons) were generated. For specific differential expression profiles the gene ontology enrichment was performed using a standard hypergeometric test with Benjamini-Hochberg multisample correction (significance at p-BH < 0.05). In each case the biological process database was used (http://geneontology.org/). All other parameters were left to default.

### Quantitative real-time PCR

Changes in expression of selected genes were validated using RNA obtained from myelinating cultures (DIV 18) grown in the presence or absence of FGF2 or F2 V2 for 24 h. Following RNA extraction, cDNA was synthesized using the Qiagen QuantiTect® Reverse Transcription Kit following the manufacturer’s instructions. Cycling parameters were as follows: first cycle (DNA wipeout step) 42 °C for 2 min, after adding reverse transcriptase, reaction buffer and primer mix second cycle: 42 °C for 20 min, then 3 min for 95 °C. Real-time PCR was performed using 1X SYBR Green master mix (Applied Biosystems), 10 ng cDNA template and 50 pmol/μl of each primer. Primers were designed using the NCBI nucleotide data base and Primer 3 software (http://biotools.umassmed.edu/bioapps/primer3_www.cgi) [67]. Primer sequences were checked with BLAST (http://blast.ncbi.nlm.nih.gov/Blast.cgi) and were purchased from IDT.

The reaction was amplified in an Applied Biosystems Fast Real-Time PCR System (ABI 7500) using the following cycle settings: 50 °C for 5 min, 95 °C for 10 min, followed by 40 cycles of 95 °C for 15 s, 60 °C for 1 min, and a final dissociation step at 95 °C for 15 s. Melt curve analysis was then performed between 75 and 99 °C in 1 °C increments. The comparative CT method (or the 2^-ΔCT^ method) [72] was used to determine differences in gene expression. For statistical analysis, a one-way ANOVA was used on the mean ΔCT for each experimental repeat to test for significant changes compared to untreated cultures and a paired t-test to test for significance in fold changes of FGF2 vs F2 V2.

### Protein assays

Mouse myelinating cultures (days in vitro - DIV18) or mouse neurosphere-derived astrocytes were cultured in the presence of 100 ng/ml FGF2 or F2 V2 and after 3 days supernatants were harvested. Protein concentrations of CXCL1 and MMP3 in the supernatants were measured using mouse CXCL1 or MMP3 Quantikine ELISA kits (both R&D Systems) according to the manufacturer’s instructions. TIMP1 protein levels were assessed by Proteome Profiler (Mouse Cytokine Array Panel A, R&D Systems), pixel densities were converted into arbitrary units using TotalLab Quant software.

### Statistics

All statistics were calculated with GraphPad Prism (GraphPad Software Inc.; La Jolla, CA, USA) using paired t-tests or one-way ANOVA (Tukey post-test unless stated otherwise) as specified in the text. A *p*-value < 0.05 was considered statistically significant, with *p* < 0.001 ***; *p* < 0.01 **; *p* < 0.05 *; n.s. = not significant.

## Results

### Astrocytes up-regulate expression of FGF2 in active multiple sclerosis lesions

**Fig. 1:**
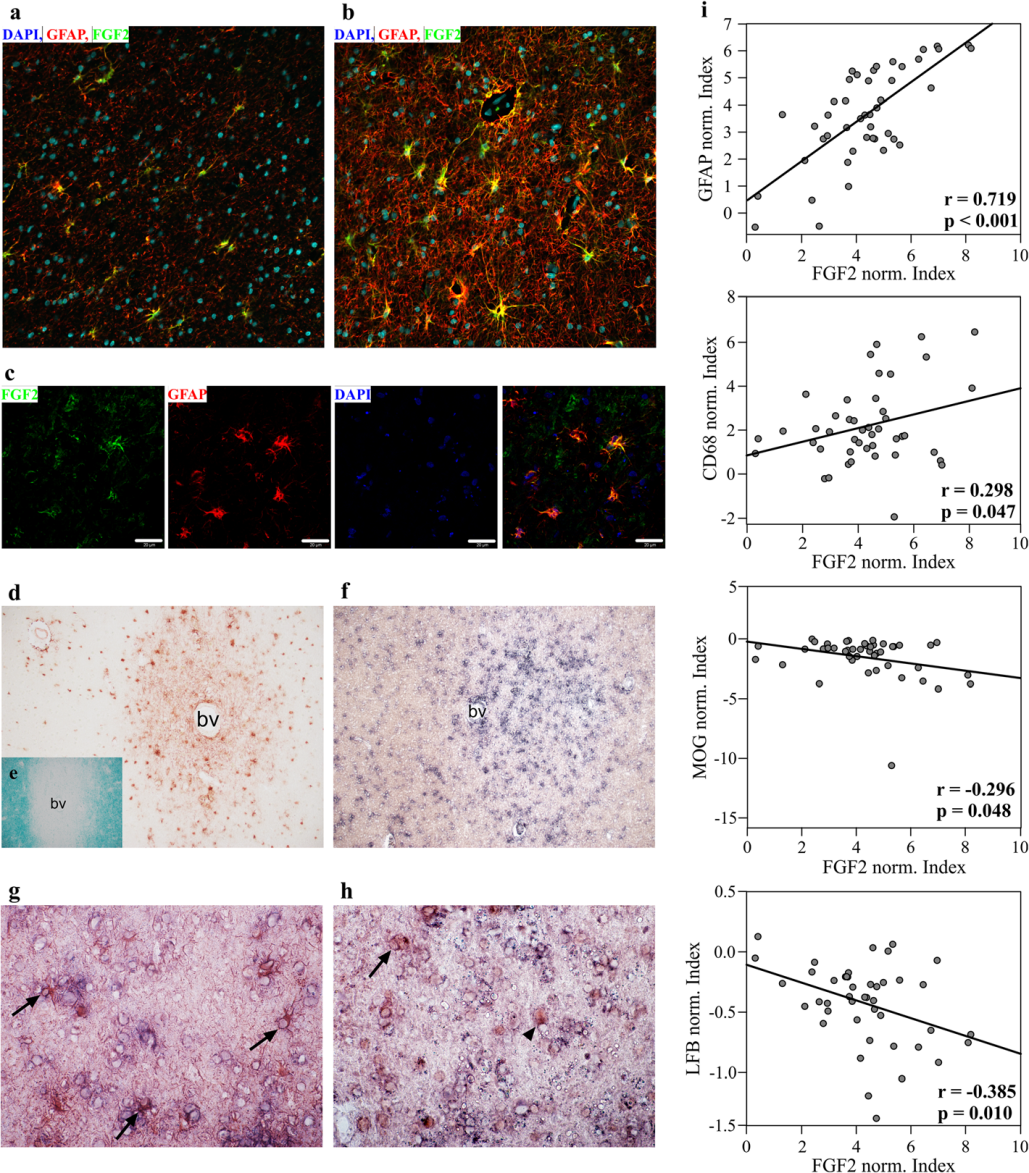
FGF2 is up-regulated in astrocytes of subcortical multiple sclerosis lesions. Fluorescence immunostaining for FGF2 and GFAP of NAWM (**a**) and active multiple sclerosis lesion (**b**) show strong FGF2 expression in the lesion. FGF2 expression co-localizes with GFAP (**c**). This finding is supported by immunohistochemistry of FGF2 expression in multiple sclerosis lesion (**d**). Demyelinating lesion is indicated by reduced Fast Luxol Blue staining around the blood vessel (**e**). In situ hybridization depicts *Fgf2* transcripts within active lesion (**f**) mainly in GFAP-positive cells (**g**, arrows), and only rarely and weakly in Olig2-positive cells (**h**, arrow). Most of the Olig2-positive cells do not express FGF2 (**h**, arrowhead). Note, ISH is weaker after immunohistochemistry. Magnifications **a**, **b**, **g** and **h**: 40x; **d-f**: 10x; bv: blood vessel, **c**: scale bars represent 20 μm. **i** Quantification of FGF2 intensity in correlation to GFAP, MOG, CD68 and LFB shows positive correlation with GFAP and inflammation (CD68) and negative correlation with myelin reactivity (LFB and MOG). Ten randomly taken images from at least 3 areas of interest per multiple sclerosis patient (*n* = 7 patients) were analyzed and normalized to the corresponding values in the NAWM of the patient. Data was log2 transformed and Pearson correlation over all cases was calculated

Expression of FGF2 was investigated in lesions and normal appearing white matter (NAWM) from seven cases of multiple sclerosis by immunohistochemistry and in situ hybridization (Fig. 1, Additional file 1). FGF2 immune reactivity was most prominent in demyelinated lesions where it was associated with extracellular staining suggestive of local release and binding of FGF2 to the extracellular matrix ([47], Fig. 1c, d). Immune fluorescence microscopy revealed these lesions contained large numbers of FGF2^+^ GFAP^+^ astrocytes, whereas only occasional FGF2^+^ astrocytes were present in NAWM. To confirm this represented de novo synthesis of FGF2 we combined in situ hybridization for *Fgf2* with immunohistochemistry for GFAP and OLIG2. *Fgf2* expression was clearly enhanced in lesions (Fig. 1f) primarily in GFAP^+^ astrocytes (Fig. 1g). However, occasional *Fgf2*^*+*^ OLIG2^+^ cells were also present (Fig. 1h); an observation in agreement with reports FGF2 can be expressed by OPC [90]. FGF2 may be expressed by some neuronal subpopulations and occasional microglia/macrophages [18, 33]. However our data are consistent with astrocytes being the dominant source of FGF2 in these lesions. This interpretation is supported by semi-quantitative analysis of immune reactivity for FGF2 and GFAP that identified a strong positive correlation between these parameters (*r* = 0.719; *p* < 0.001) (Fig. 1i). To determine if FGF2 expression also correlated with inflammatory activity and/or demyelination, immune reactivity for CD68 was used as a proxy for inflammation and LFB histochemistry and MOG immune reactivity were used to assess demyelination. This revealed FGF2 immune reactivity correlated positively with inflammation (CD68 immune reactivity: *r* = 0.298, *p* = 0.047) and negatively with myelination (MOG immune reactivity: *r* = − 0.296, *p* = 0.048; LFB histochemistry: *r* = − 0.385, *p* = 0.010) (Fig. 1i). Inflammatory demyelination in MS is therefore associated with increased expression of FGF2 by astrocytes.

### Restricting signal transduction to FGFR1 dissociates inhibition of myelination by FGF2 from its mitogenic potential

Having confirmed FGF2 was available to influence lesion development we then explored its pathophysiological significance in tissue culture. As stated previously FGF2 is pleiotropic and in addition to its detrimental effects on oligodendrocyte differentiation and (re) myelination [25, 79] [53] is neuroprotective in EAE [66, 68]. The mechanistic basis of this neuroprotective response is poorly understood but may include effects that enhance neurogenesis, proliferation, mobilization and recruitment of OPC, and/or limit immune cell recruitment across the blood brain barrier [3, 66, 68].

As previous studies indicate the mitogenic potential of FGF2 is FGFR1 dependent [25] we investigated the effects of F2 V2, a novel FGFR1-selective agonist, on oligodendrogenesis and myelination in cultures derived from embryonic spinal cord; an in vitro model that replicates the cellular complexity of the CNS and allows identification of direct and “off target” effects on OPC biology [51]. F2 V2 was generated by deleting the N-terminal 26 amino acids of FGF2, a domain that contributes to promiscuity of receptor recognition by other FGF family members [7, 41]. In the case of FGF2 this deletion generated a mutant that retained the ability of the native protein to activate FGFR1, but reduced its ability to induce FGFR2-, FGFR3- and FGFR4-dependent proliferation in FGFR transfected reporter cells by approximately 90% (Additional file 2).

**Fig. 2:**
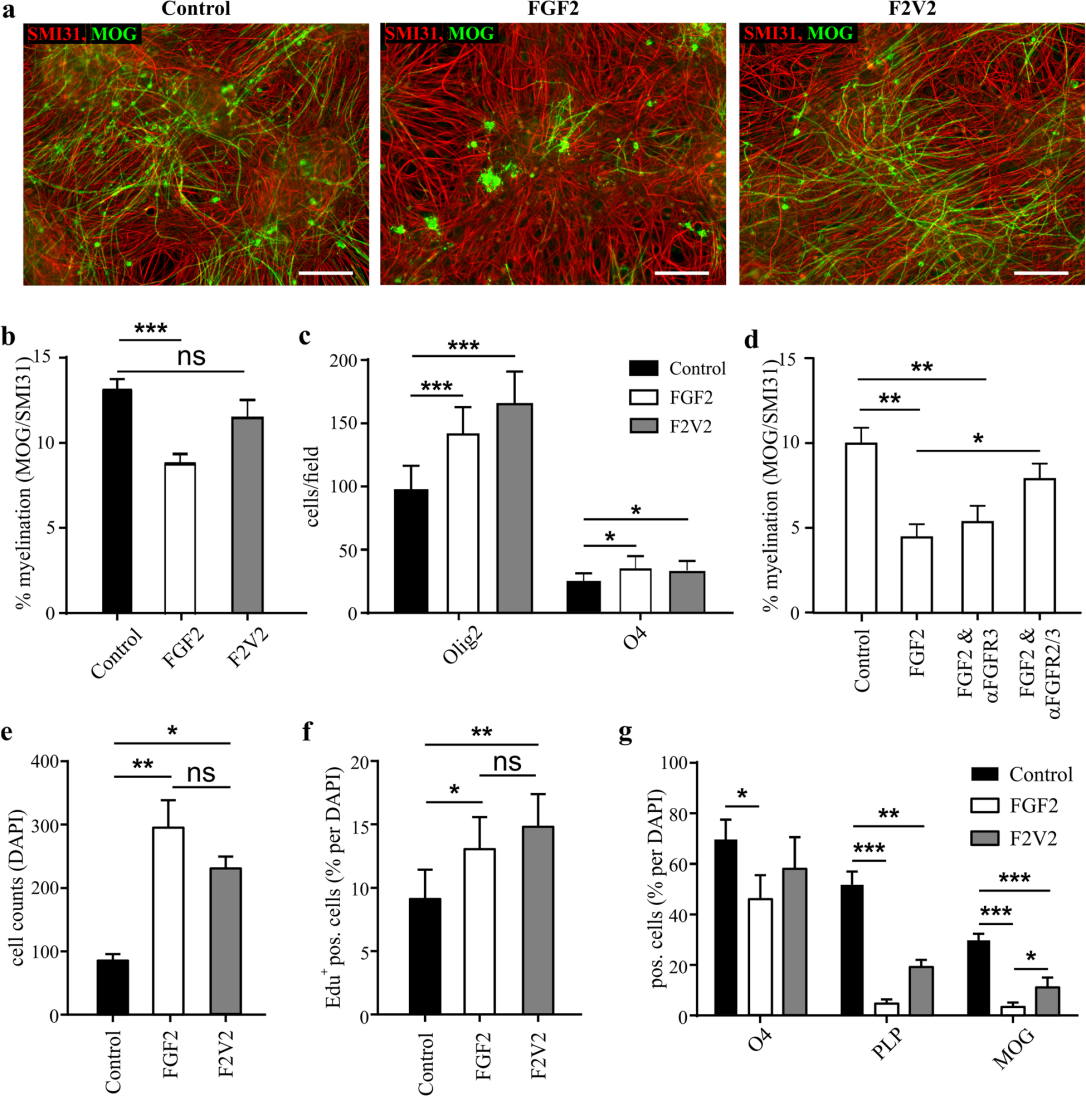
N-terminal truncation of FGF2 abrogates its ability to inhibit myelination without disrupting its mitogenic activity*.* **a** Myelinating rat CNS cultures were treated with 100 ng/ml FGF2 or F2 V2 for 10 days (DIV18–28); provided are representative images demonstrating the differential effects of FGF2 and F2 V2 on myelination rates determined by MOG pos. sheets. Bars represent 100 μm. **b** In cultures treated with FGF2 or F2 V2 for 10 days FGF2, but not F2 V2 significantly inhibits myelination as determined by percentage of MOG pos. sheets/ SMI31 (one-way ANOVA). **c** Both FGF2 and F2 V2 significantly increase the number of Olig2^+^ and O4^+^ cells (one-way ANOVA). **d** Blocking antibodies directed towards FGFR3 (PRO-001) or towards FGFR2/3 (PRO-007) have different effects on the inhibition of myelination by FGF2, with blocking FGFR2/3 abrogating the effect of FGF2 (one-way ANOVA, *p* < 0.01 for FGF2 vs FGF2 + αFGFR2/3). **e**, **f** Proliferation of immunopurified A2B5^+^ OPCs in the presence or absence of FGF2 or F2 V2 (100 ng/ml). **e** Both FGF2 and F2 V2 act as strong mitogens on purified OPCs cultured for 6 days in differentiation media (modified Sato’s medium) as cell numbers (DAPI staining) significantly (one-way ANOVA) increase 3.4 or 2.7 times after treatment with FGF2 or F2 V2 respectively. **f** A2B5^+^ progenitors were maintained in modified Sato’s medium in the presence of FGF2 or F2 V2 for 72 h and proliferation was determined by EdU incorporation. There is no significant difference in the ability of FGF2 and F2 V2 to maintain OPC proliferation (one-way ANOVA). **g** A2B5^+^ progenitors were cultured for 6 days in modified Sato’s medium and the amount of O4^+^, PLP^+^ or MOG^+^ pos. cells (% of DAPI) was quantified. FGF2 treatment significantly reduced O4 expression (*p* < 0.05, one-way ANOVA) and almost abolished PLP and MOG expression (*p* < 0.001 for both markers, one-way ANOVA). Data are presented as means ± SEM from at least 3 independent experiments; * *p* < 0.05; ** *p* < 0.01; *** *p* < 0.001; ns – not significant

In myelinating cultures FGF2 and F2 V2 induced comparable increases in OLIG2^+^ and O4^+^ cell numbers indicating these effects were FGFR1-dependent, but only FGF2 inhibited myelination (Fig. 2a-c). Activation of FGFR1 is therefore sufficient to drive OPC proliferation and initiate oligodendrocyte differentiation but in isolation has no significant effect on myelination. This implies inhibition of myelination by FGF2 is dependent on signal transduction via FGFR2 and/or FGFR3 (expression of FGFR4 in the CNS is minimal [90];). This was confirmed using FGFR3- and FGFR2/3-specific neutralizing antibodies [25, 62, 80]. FGF2-mediated inhibition of myelination was unaffected by the FGFR3-specific antibody, but was abrogated significantly by the FGFR2/FGFR3-specific reagent demonstrating inhibition of myelination by FGF2 in this model system is FGFR2-dependent (Fig. 2d).

We then compared the effects of FGF2 and F2 V2 on oligodendrogenesis in the absence of other cell types using immunopurified A2B5^+^ progenitors (Fig. 2e-g). The mitogenic potential of FGF2 and F2 V2 was virtually identical (Fig. 2e, f), but unlike the situation in myelinating cultures they were unable to expand the number of O4^+^ oligodendrocytes, an effect associated with reduced differentiation of OPC’s into more mature PLP^+^ and MOG^+^ oligodendrocytes (Fig. 2g). This suggests FGFR1 signaling in myelinating cultures is not only mitogenic for OPC, but also induces an “off-target” response that allows them to drop out of cell cycle and differentiate into O4^+^ oligodendrocytes, a necessary step towards successful (re)myelination.

### OPC proliferation and inhibition of myelination are associated with distinct FGFR-specific transcriptional profiles

**Fig. 3:**
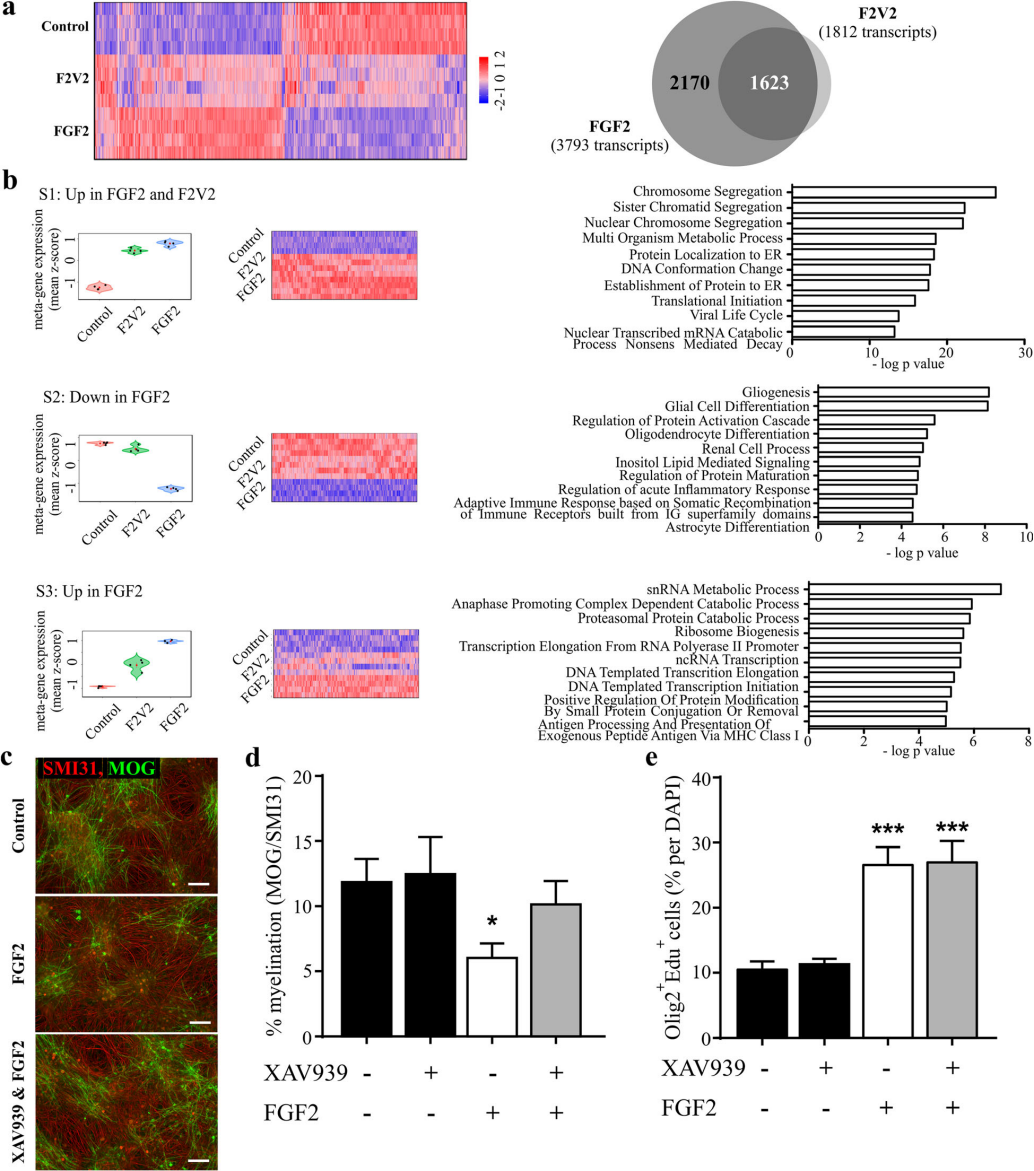
Transcriptional profiling identifies a fingerprint associated with FGF2-mediated inhibition of myelination, which is dependent on Wnt signaling. Myelinating rat CNS cultures were treated with 100 ng/ml FGF2 or F2 V2 for 24 h at DIV18 and gene expression was analyzed by performing an Affymetrix rat gene array. **a** Hierarchical cluster analysis of the 9637 transcripts with FDR-adjusted *p* < 0.05 for any of the treatments (FGF2 or F2 V2) was performed. Shown are expression values for the four technical replicates in each group; gene expression level is represented by colour intensity (high expression in red, low expression in blue). Expression values were scaled using Z-score. 3793 or 1812 transcripts were differentially regulated by FGF2 or F2 V2 (fold change > + 1.4; FDR-adjusted *p* < 0.05), in a comparative analysis of these genes 1623 transcripts are regulated in a similar manner by FGF2 (dark grey intersection) and 2170 are regulated selectively by FGF2. **b** Three distinct transcriptional profiles are plotted, with a subset of 1582 transcripts significantly up-regulated by both FGF2 and F2 V2 (top panel – Additional file 7: Table S1), 486 transcripts specifically down-regulated by FGF2 but not regulated by F2 V2 (middle panel – Additional file 7: Table S2) and 1026 transcripts specifically more up-regulated by FGF2 (lower panel – Additional file 7: Table S3). Shown are the meta-gene expression, heatmaps and 10 most significantly enriched GO terms for each of the three signature profiles. This revealed a mitogenic profile in the genes up-regulated by both FGF2 and F2 V2 (Additional file 7: Table S1), a signature for glia cell differentiation in the genes specifically down-regulated by FGF2 (Additional file 7: Table S2) and a profile identifying pathways potentially contributing to the inhibition of myelination by FGF2 (Additional file 7: Table S3). Myelinating rat CNS cultures were treated with 100 ng/ml FGF2 in the absence or presence of a specific inhibitor of the Wnt pathway (20 μM Tankyrase Inhibitor XAV939) for 10 days at DIV18 and myelination rates (MOG positive myelin sheets) were analyzed (**c**, **d**) or proliferation of oligodendrocyte lineage cells was analyzed after 3 days of treatment by incorporation of EDU in Olig2 positive cells (**e**). **c** Representative images for untreated (Control) cultures or cultures treated with FGF2 or FGF2 & XAV939 are shown, scale bars represent 100 μm. (**d**, **e**) shown are mean + SEM from at least 4 independent experiments and *p*-values for one-way ANOVA as compared to control; * *p* < 0.05; ** *p* < 0.01; *** *p* < 0.001

To identify how FGF2 and F2 V2 induce these FGFR-dependent effects on OPC proliferation/differentiation and myelination we performed a gene microarray study in myelinating cultures. Hierarchical cluster analysis of 9637 transcripts with significant changes in expression induced by either ligand (FDR-adjusted *p* < 0.05) revealed a considerable overlap in the transcriptional response to the two ligands (Fig. 3a). FGF2 and F2 V2 differentially regulated 3793 (1983 up; 1810 down) and 1812 (1089 up; 723 down) transcripts, respectively (fold change ≥ +/− 1.4; Fig. 3a; Additional file 3). We reasoned the 1623 transcripts differentially regulated by both FGF2 and F2 V2 define a FGFR1-dependent transcriptome that would reflect these ligands’ ability to enhance OPC proliferation. This interpretation was supported by differential expression profile and gene ontology enrichment analysis which revealed the most highly enriched GO terms associated with FGFR1 signaling were related to cell cycle and DNA replication (Fig. 3b, Additional file 7: Table S1). Conversely, we reasoned the 2170 transcripts only regulated in response to FGF2 defined a transcriptome containing components responsible for FGFR2-dependent inhibition of myelination (Additional file 3). This was also supported by gene ontology enrichment analysis which demonstrated this “non-FGFR1 associated” transcriptome was enriched in GO terms related to glial differentiation including “Gliogenesis”, “Glial Cell Differentiation”, “Astrocyte Differentiation” and “Oligodendrocyte Differentiation” (Fig. 3b).

### FGFR2 mediated inhibition of myelination is Wnt dependent

To identify pathways contributing to FGFR2-dependent inhibition of myelination we then focused on a third transcriptional profile consisting of those genes more up-regulated by FGF2 than F2 V2 (Fig. 3b, Additional file 7: Table S3). This profile was significantly enriched in 39 GO terms including three associated with Wingless (Wnt) signaling (Additional file 4 and Additional file 7). This was significant as Wnt signaling not only contributes to developmental control of myelination, but is also implicated in failure of remyelination in MS [23, 49, 75]. We therefore performed an additional analysis to test this association using an alternative software package (Partek Genomics Suite and Partek Pathway version 6.6). This identified 1047 transcripts differentially regulated by FGF2 relative to F2 V2 (528 down, 519 up; FDR-adjusted *p* < 0.05 and +/− 1.4 fold change). These transcripts were significantly enriched in 44 KEGG pathways that again included Wnt signaling (Additional file 5). Validation of our array data for selected components of the Wnt signaling pathway by qPCR (Additional file 6) provided additional evidence that inhibition of myelination by FGF2 was associated with Wnt signaling. We therefore tested the effect of disrupting Wnt signaling on FGF2-mediated inhibition of myelination using XAV939, a tankyrase 1 and 2 inhibitor that disrupts Wnt signaling by stabilizing the β-catenin destruction complex. XAV939 abrogated the ability of FGF2 to inhibit myelination (Fig. 3d; FGF2 versus FGF2 + XAV939, *p* < 0.05; Newman-Keuls post-test) demonstrating inhibition of myelination by FGF2 is dependent on activation of Wnt signaling. In contrast, XAV939 had no effect on basal levels of myelination, nor did it influence FGF2 induced (FGFR1-dependent) OLIG2^+^ cell proliferation (Fig. 3c-e).

### FGFR1 supports induction of pro-myelinating and neuroprotective factors

Having demonstrated the “FGFR1-associated” F2 V2 transcriptome was enriched in pathways associated with cell cycle and DNA replication (Fig. 3b), we explored this data set for evidence it might also support OPC differentiation or mediate other potentially neuroprotective effects. We identified multiple transcripts encoding products with pro-myelinating and/or immunomodulatory effects including leukaemia inhibitory factor (LIF [76];), interleukin 11 (IL11 [38, 91];); heparin-binding EGF-like growth factor (HB-EGF [1, 13, 59];), chemokine (C-X-C motif) ligand 1 (CXCL1 [56];), tissue inhibitor of metalloproteinase 1 (TIMP1 [55];) and Cluster of Differentiation 93 (CD93 [40];) (Table 1). Conversely, F2 V2 was unable to replicate the ability of FGF2 to up-regulate expression of matrix metalloproteinases (MMP) contributing to blood–brain barrier breakdown in inflammatory and ischemic disorders [65, 89] (*Mmp13*: FGF2 fold change + 28; *Mmp3*: FGF2 fold change + 6.8; F2 V2 fold change < +/− 1.2 for both genes) (Table 1).

**Table 1:** qPCR validation for immunoregulatory/neuroprotective genes

Gene	qPCR validation fold change (*p*-value versus control)		Microarray fold change		Primer sequence
	FGF2	F2 V2	FGF2	F2 V2	
*Cd93*	240.2 ± 101.2 (***)	44.9 ± 9.1 (***)	74.1	23.8	CATCTCACTCTTGCTGGCTCT TCTCCTCTTTCTTGGCTTTCC
*Il11*	48.4 ± 19.8 (***)	14.9 ± 3.7 (**)	47.5	6.3	CTCCCCTCGAGTGTCTTCAG CCATCAGCTGGGAATTTGTC
*Hb-egf*	22.2 ± 12.0 (***)	7.1 ± 2.5 (**)	11.9	4.9	TTTCTCCTCCAAGCCACAAG TTCCTCTTCTTTTTCCCGTTC
*Lif*	13.6 ± 4.6 (***)	3.8 ± 1.7 (*)	19.4	4.0	CCTTCCCATCACCCCTGT CGTTGAGTTGAGCCAGTTGA
*Cxcl1*	12.5 ± 4.1 (***)	8.4 ± 1.8 (***)	4.0	5.6	AACCGAAGTCATAGCCACACTC CACCCTTTAGCATCTTTTGGAC
*Timp1*	6.2 ± 0.9 (**)	5.8 ± 1.8 (**)	2.8	2.3	CTGGTTCCCTGGCATAATCT ATCGCTCTGGTAGCCCTTCT
*Mmp13*	28.6 ± 14.0 (***)	2.2 ± 1.8 (ns)	28.1	1.2	CTGCGGTTCACTTTGAGGA GAGGCGGGGATAGTCTTTGT
*Mmp3*	8.1 ± 3.5 (*)	0.4 ± 1.2 (ns)	6.8	−1.0	CCCGTTTCCATCTCTCTCAA GACATCAGGGGATTCTGTGG

Shown are mean +/− SEM of at least 5 independent experiments; *p*-values for ΔCt of Control vs FGF2 or F2 V2 respectively (one-way ANOVA, Holm-Sidak post-test); * *p* < 0.05, ** *p* < 0.01, *** *p* < 0.001, ns - not significant

**Fig. 4:**
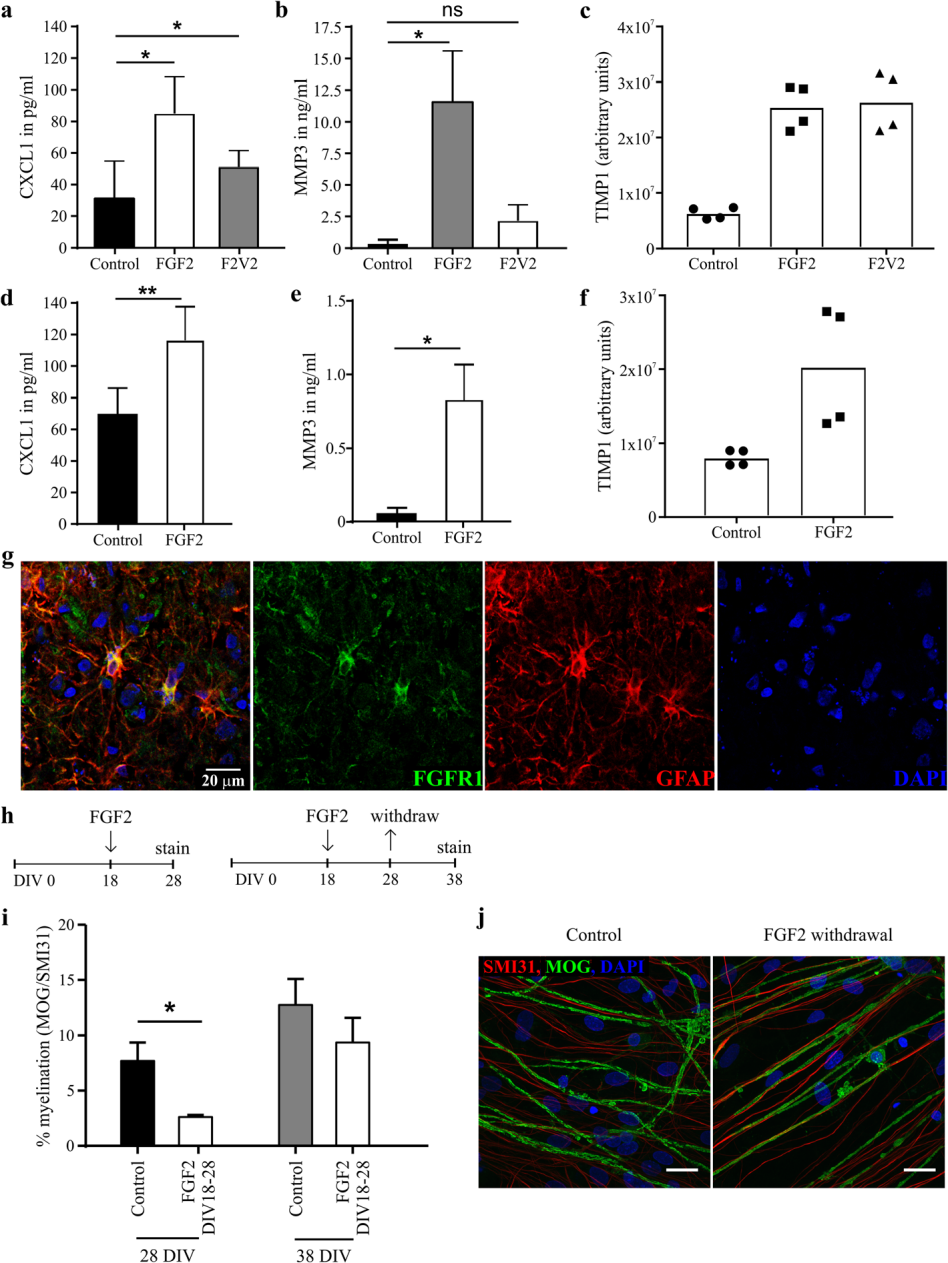
FGFR1 supports induction of pro-myelinating and neuroprotective factors. Mouse myelinating cultures were treated with 100 ng/ml FGF2 or F2 V2 at DIV18 for 3 days and protein levels in the cell culture supernatant were analyzed by ELISA for CXCL1 (**a**) and TIMP1 (**b**) or by Proteom Profiler (**c**, Mouse Cytokine Array Panel **a**). **a**, **b** FGF2 treatment results in a significant increase in protein levels for both CXCL1 and MMP3 (*p* < 0.05), whereas F2 V2 only significantly increased the levels of CXCL1. Data are presented as means + SEM from 6 independent experiments; * *p* < 0.05. **c** TIMP1 levels are increased in both FGF2 and F2 V2 treated cultures; data are presented as technical duplicates from 2 independent experiments, pixel densities are represented as arbitrary units. **d-f** A monolayer of neurosphere-derived mouse astrocytes was treated with 100 ng/ml recombinant FGF2 for 3 days and concentrations of CXCL1 (**d**), MMP3 (**e**) or TIMP1 (**f**) were determined by ELISA (**d**, **e**) or Proteom Profiler (**f**, Mouse Cytokine Array Panel **a**). **d**, **e** Data are presented as means ± SEM from 4 independent experiments; * *p* < 0.05; ** *p* < 0.01 (paired t-test), **f** Data are presented as technical duplicates from 2 independent experiments, arbitrary units represent pixel densities. **g** FGFR1 expression co-localizes with GFAP^+^ astrocytes in active multiple sclerosis. **h**, **i** Myelinating CNS cultures were treated in the absence or presence of 100 ng/ml FGF2 for 10 days from DIV 18 onwards and either directly fixed and stained for MOG / SMI31 after a total of 28 days (28 DIV) or after 38 days in vitro (38 DIV); shown are mean + SEM from at least 4 independent experiments and p-values for paired t-test; * *p* < 0.05. **j** Representative confocal immunofluorescence images (63x magnification, Maximum Intensity Projection) of 38 DIV culture demonstrating withdrawal of FGF2 (18 DIV – 28 DIV) is followed by rapid remyelination resulting in the formation of myelinated internodes that are indistinguishable from those in untreated cultures. Scale bars represent 10 μm

These observations were validated at the protein level by assaying CXCL1, MMP3 and TIMP1 in supernatants from mouse myelinating cultures (Fig. 4a-c). F2 V2 retained the ability of FGF2 to increase CXCL1 and TIMP1 levels confirming these are at least in part FGFR1-dependent responses, but induced no significant increase in MMP3 confirming this response is FGFR1-independent.

Previous studies suggest astrocytes were a potential source of these proteins [16, 55, 70]. We therefore analyzed supernatants harvested from mouse astrocytes cultured in the presence or absence of FGF2 (Fig. 4d-f). FGF2 increased CXCL10 and TIMP1 concentrations to levels comparable to that seen in myelinating cultures indicating astrocytes are the major source of these proteins. The ability of astrocytes to mediate these FGFR1-associated responses in MS is supported by our demonstration astrocytes in MS lesions are FGFR1^+^ (Fig. 4g). FGF2 treated astrocytes also secreted low levels of MMP3, but its concentration was an order of magnitude lower than in supernatants from myelinating cultures indicating that the major source in these cultures is some other cell type (Fig. 4e).

Taken together these observations suggest enhancing FGFR1-dependent responses whilst suppressing signal transduction by FGFR2 would enhance lesion repair, but for this to be effective the detrimental effects of FGF2 on (re)myelination must be reversible. We therefore investigated the effects of withdrawing FGF2 from myelinating cultures after 10 days treatment (100 ng/ml; DIV 18–28). Quantifying myelination 10 days later (DIV 38) revealed withdrawal of FGF2 was followed by rapid myelination that resulted in levels of ensheathment comparable to that seen in untreated control cultures (Fig. 4h-j).

## Discussion

To investigate how FGF2-dependent mechanisms contribute to lesion development in MS we first mapped its expression in lesions and NAWM by in situ hybridization and immunohistochemistry. This combination of techniques revealed astrocytes up-regulate expression of FGF2 in active lesions and the magnitude of this response correlated with lesion activity as defined by CD68 immune reactivity. This identified astrocytes as a major source of FGF2 in active lesions, although activated microglia/macrophages may also contribute to this response [18], and provides a logical explanation why CSF concentrations of FGF2 correlate with disease activity [69, 77].

We then went on to explore how increasing FGF2 availability may influence lesion development, focusing on understanding how this single mediator not only modulates oligodendrogenesis and myelination, but also mediates neuroprotection [3, 66, 68]. Previous studies provide compelling evidence this is determined at least in part by cell specific differences in FGFR expression [4, 25]. We therefore investigated the effect of restricting signaling to FGFR1 in myelinating cultures that replicate the cellular complexity of the CNS parenchyma in which FGFR1 is expressed by multiple cell types (astrocytes > > OPC > neurons > microglia) [90]. This strategy not only dissociated the mitogenic potential of FGF2 from its inhibitory effects on myelination, but also provided a tool that allowed us to define how specific FGFRs contribute to the functional outcome of FGF2 signaling in the CNS. This revealed inhibition of myelination by FGF2 is dependent on FGFR2 mediated activation of Wnt signaling; pharmacological inhibition of Wnt signaling being sufficient to suppress inhibition of myelination by FGF2. It should be noted inhibiting Wnt signaling had no significant effect on progenitor cell proliferation, which is in agreement with our observation this is driven primarily via activation of FGFR1. Crosstalk between FGF and Wnt signaling is well documented in other settings [45, 83, 86] and we suspect this occurs at the level of GSK3β, a major component of the β-catenin destruction complex, which is modulated in response to FGFR-mediated activation of PI3K/Akt signaling [46]. We anticipate this is an oligodendrocyte intrinsic response as terminally differentiated oligodendrocytes not only express high levels of FGFR2, but Wnt signaling is also activated in cells of the oligodendrocyte lineage in MS lesions [23]. However, we have not formally excluded the possibility “off target” effects triggered by FGFR2 mediated activation of Wnt in other cells contribute to inhibition of myelination by FGF2. Additional studies using human oligodendrocytes are therefore required to validate this hypothesis and to determine the relative importance of downstream pathways associated with FGFR signal transduction, in particular extracellular signal-regulated protein kinase (ERK)/mitogen-activated protein kinase (MAPK) pathway and PI3K/Akt/mechanistic target of rapamycin (mTOR) pathways which are known to modulate oligodendrocyte differentiation and myelination [20, 29, 30, 32, 36, 44].

These observations are important as they identify FGF2 as a factor up-regulated in active MS lesions that disrupts myelination via activation of Wnt, a pathway which plays important roles in regulating myelination and remyelination, and is dysregulated in MS [23, 37, 82]. However whilst FGFR2 dependent inhibition of (re)myelination may be one biological outcome of increasing FGF2 availability in the CNS, this is accompanied by a host of FGFR1-dependent responses predicted to enhance lesion repair. Activation of FGFR1 is not only directly mitogenic for OPC, but also induces expression of pro-myelinating and immunomodulatory factors by other cell types. These include pro-myelinating factors predicted to promote OPC proliferation, migration and/or survival (IL-11, LIF, CXCL1, TIMP1, HB-EGF) and others with immunomodulatory and/or neuroprotective properties (CD93, IL-11, LIF; HB-EGF) predicted to restrain inflammatory activity in the CNS [13, 24, 38, 42, 55, 56, 64, 91]. Conversely, skewing signaling to favor FGFR1 also prevents the ability of FGF2 to induce MMPs implicated in blood-brain barrier damage and leucocyte recruitment in multiple sclerosis and other neurological diseases [31, 65, 88, 89].

We propose this concatenation of FGFR1-dependent effects are responsible for the neuroprotective potential of FGF2 in EAE [66], but the relative importance of FGFR1-dependent responses in different cell type remains unclear. FGFR1 expression in oligodendrocytes is reported to suppress disease activity in EAE [61] and enhance remyelination following chronic cuprizone mediated demyelination [92]. However these interpretations should be treated with caution as tamoxifen was used to ablate expression of FGFR1 in these studies. Tamoxifen not only suppresses disease activity in EAE [6], but also accelerates repair of demyelinated lesions in vivo [34]. Future studies investigating the functional significance of cell type specific activation of FGFR1 signaling pathways in animal models of MS must take these confounding issues into account.

To the best of our knowledge this is the first demonstration an FGFR1-specific agonist can be used to uncouple the detrimental effects of FGF2 on myelination from its ability to induce a broadly neuroprotective signaling environment. This raises the interesting possibility, that CNS delivery of FGFR1 specific agonists such as F2 V2 may provide a novel strategy to induce neuroprotection without compromising (re)myelination. A concept consistent with expression of FGFR1 by astrocytes, Olig2^+^ progenitors and occasional neurons in the CNS ([17, 90], and this study), although selective delivery of therapeutic agents into the CNS remains a major challenge [5].

In summary we demonstrate FGF2 expression is up-regulated by astrocytes in inflammatory MS lesions where it may contribute to remyelination failure, whilst simultaneously providing a neuroprotective signaling environment. These diametrically opposed functions can be uncoupled by polarizing FGFR activation to favor FGFR1 which retains the mitogenic activity of FGF2 and its ability to induce expression of neuroprotective, pro-myelinating and anti-inflammatory factors in the absence of a detrimental effect on myelination. We propose FGFR1 selective agonists may provide a generalized strategy to reduce tissue damage and accelerate lesions repair in inflammatory and ischaemic CNS diseases.

## Supplementary information

**Additional file 1. Online Resource 1:** MS patient data and tissue used

**Additional file 2. Online Resource 2:** N-terminal deletion of FGF2 results in selective activation of FGFR1.

**Additional file 3. Online Resource 3:** Differential regulation of myelin by FGF2 and F2 V2.

**Additional file 4. Online Resource 4:** Full list of enriched GO terms for genes more up-regulated by FGF2.

**Additional file 5. Online Resource 5:** Pathways of genes differentially regulated in FGF2 vs F2 V2.

**Additional file 6. Online Resource 6:** qPCR validation for Wnt pathway genes.

**Additional file 7.** Microarray data.
