# Supplementary material for: Polarizing receptor activation dissociates fibroblast growth factor 2 mediated inhibition of myelination from its neuroprotective potential
Source: Acta Neuropathol Commun. 2019 Dec 19;7:212. doi: 10.1186/s40478-019-0864-6 (PMC6923900; doi:10.1186/s40478-019-0864-6)
Supplement: Supplementary file 1 — Additional file 1. Online Resource 1: MS patient data and tissue used [file 40478_2019_864_MOESM1_ESM.pdf]

Online Resource 1: MS patient data and tissue used

Patient	Tissue block	Age (yr)	Sex	Disease duration (yr)	pmd (hr)	Disease course	Cause of death	ISH	qIHC			IF
									NAWM	RM	Lesion	
MS12	P3B2	56	f	31	8	SP	Breast carcinoma					X
MS40	A1D3	58	f	21	6	PP	Bronchopneumonia		1	2	2	
MS46	A5C4	40	m	23	18	SP	Multiple Sclerosis					X
MS54	P4D3	69	f	31	11	SP	Acute pyelonephritis					X
MS71	A2C4	78	f	42	5	SP	Metastatic carcinoma of bronchus					X
MS79	P4B3	49	f	21	7	SP	Bronchopneumonia		1	2	2	X
MS106	P5D6	39	f	21	18	PP	Bronchopneumonia		1	2	3	X
MS122	A2E7	44	m	Unknown	16	SP	Bronchopneumonia		1	2	2	
	P2A4							X	1		4	
MS136	P5B2	40	m	9	10	SP	Respiratory failure		1		4	
	P5C4							X	1		4	
MS154	P5B7	34	f	Unknown	12	SP	Bronchopneumonia		1		3	
	P5C5								1	3	6	
MS176	P1A2	37	m	27	12	PP	Intestinal obstruction		1		5	
MS387	A4B5	42	f	11	13	SP	Multiple Sclerosis					X

f female; m male; PP primary progressive multiple sclerosis; SP secondary progressive multiple sclerosis
