# Supplementary material for: Polarizing receptor activation dissociates fibroblast growth factor 2 mediated inhibition of myelination from its neuroprotective potential
Source: Acta Neuropathol Commun. 2019 Dec 19;7:212. doi: 10.1186/s40478-019-0864-6 (PMC6923900; doi:10.1186/s40478-019-0864-6)
Supplement: Supplementary file 2 — Additional file 2. Online Resource 2: N-terminal deletion of FGF2 results in selective activation of FGFR1. [file 40478_2019_864_MOESM2_ESM.pdf]

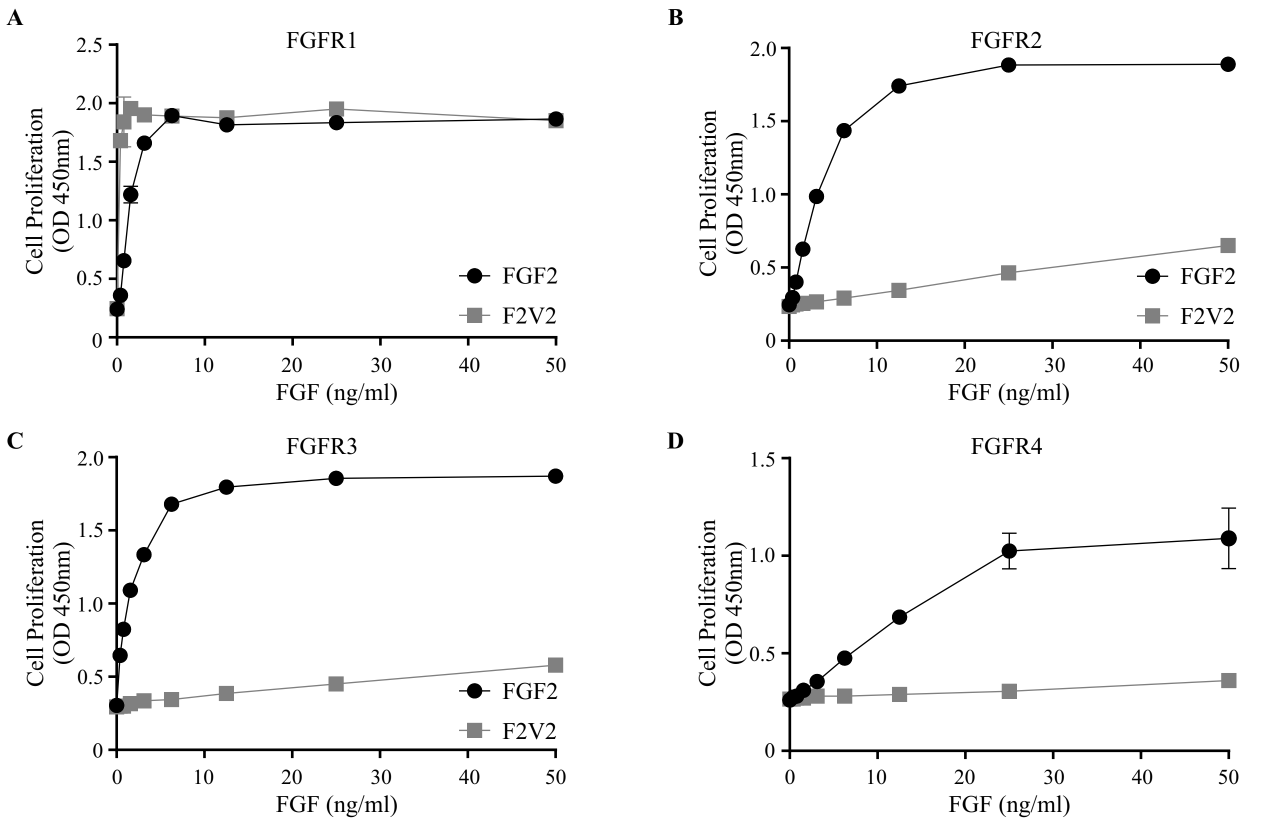

Online Resource 2. N-terminal deletion of FGF2 results in selective activation of FGFR1.
Proliferation of FDCP-1 cells transfected with FGFR1 (**A**), FGFR2 (**B**), FGFR3 (**C**) or FGFR4 (**D**) following incubation with FGF2 or with a 26 amino acid N-terminal truncated variant of FGF2 (F2V2).
