# Supplementary material for: Polarizing receptor activation dissociates fibroblast growth factor 2 mediated inhibition of myelination from its neuroprotective potential
Source: Acta Neuropathol Commun. 2019 Dec 19;7:212. doi: 10.1186/s40478-019-0864-6 (PMC6923900; doi:10.1186/s40478-019-0864-6)
Supplement: Supplementary file 3 — Additional file 3. Online Resource 3: Differential regulation of myelin by FGF2 and F2 V2. [file 40478_2019_864_MOESM3_ESM.pdf]

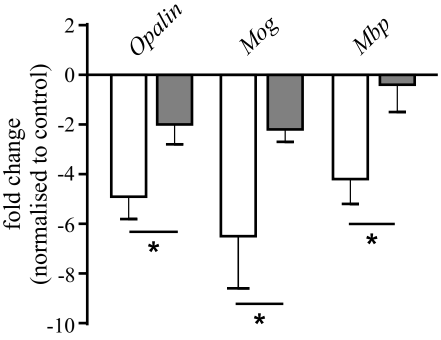

gene	Primer sequence
Opalin	ACCTTCTTGAGCACCTCGTG<br>TGATCCAGCGAAGAAGAACC
Mog	GCAGGTCTCTGTAGGCCTTG<br>GGCACTCTCAGGAAGTGAGG
Mbp	TACCCTGGCTAAAGCAGAGCA<br>GGGAGCCGTAGTGGGTAGT

Online Resource 3. Differential regulation of myelin by FGF2 and F2V2.
qPCR validation of some genes of interest from the microarray (paired t-test for fold changes FGF2 vs F2V2). Data are presented as means ± SEM from at least 4 independent experiments; * $p<0.05$; ** $p<0.01$
