# Supplementary material for: Polarizing receptor activation dissociates fibroblast growth factor 2 mediated inhibition of myelination from its neuroprotective potential
Source: Acta Neuropathol Commun. 2019 Dec 19;7:212. doi: 10.1186/s40478-019-0864-6 (PMC6923900; doi:10.1186/s40478-019-0864-6)
Supplement: Supplementary file 4 — Additional file 4. Online Resource 4: Full list of enriched GO terms for genes more up-regulated by FGF2. [file 40478_2019_864_MOESM4_ESM.pdf]

Online Resource 4: full list of enriched GO terms for genes more up-regulated by FGF2 (“S3” in Figure 3B)

GO TERM	ENRICHMENT P-VALUE
SNRNA_METABOLIC_PROCESS	1.05E-07
ANAPHASE_PROMOTING_COMPLEX_DEPENDENT_CATABOLIC_PROCESS	1.20E-06
PROTEASOMAL_PROTEIN_CATABOLIC_PROCESS	1.41E-06
RIBOSOME_BIOGENESIS	2.42E-06
TRANSCRIPTION_ELONGATION_FROM_RNA_POLYMERASE_II_PROMOTER	3.02E-06
NCRNA_TRANSCRIPTION	3.10E-06
DNA_TEMPLATED_TRANSCRIPTION_ELONGATION	5.26E-06
DNA_TEMPLATED_TRANSCRIPTION_INITIATION	6.79E-06
POSITIVE_REGULATION_OF_PROTEIN_MODIFICATION_BY_SMALL_PROTEIN_CONJUGATION_OR_REMOVAL	9.73E-06
ANTIGEN_PROCESSING_AND_PRESENTATION_OF_EXOGENOUS_PEPTIDE_ANTIGEN_VIA_MHC_CLASS_I	1.05E-05
REGULATION_OF_PROTEIN_UBIQUITINATION_INVOLVED_IN_UBIQUITIN_DEPENDENT_PROTEIN_CATABOLIC_ PROCESS	1.22E-05
REGULATION_OF_LIGASE_ACTIVITY	1.31E-05
NEGATIVE_REGULATION_OF_PROTEIN_MODIFICATION_BY_SMALL_PROTEIN_CONJUGATION_OR_REMOVAL	1.72E-05
POSITIVE_REGULATION_OF_LIGASE_ACTIVITY	1.87E-05
PROTEIN_FOLDING	2.27E-05
TRANSCRIPTION_INITIATION_FROM_RNA_POLYMERASE_II_PROMOTER	2.71E-05
TRANSLATIONAL_ELONGATION	2.80E-05
REGULATION_OF_ESTABLISHMENT_OF_PLANAR_POLARITY	3.19E-05
**NON_CANONICAL_WNT_SIGNALING_PATHWAY**	**3.83E-05**
REGULATION_OF_CELLULAR_AMINO_ACID_METABOLIC_PROCESS	3.96E-05
RNA_SPLICING_VIA_TRANSESTERIFICATION_REACTIONS	4.73E-05
REGULATION_OF_CELLULAR_PROTEIN_CATABOLIC_PROCESS	6.42E-05
MITOCHONDRIAL_TRANSLATION	7.33E-05
NIK_NF_KAPPAB_SIGNALING	7.95E-05
TRANSLATIONAL_TERMINATION	8.88E-05
ANTIGEN_PROCESSING_AND_PRESENTATION_OF_PEPTIDE_ANTIGEN_VIA_MHC_CLASS_I	1.06E-04
**POSITIVE_REGULATION_OF_CANONICAL_WNT_SIGNALING_PATHWAY**	**1.07E-04**
**NEGATIVE_REGULATION_OF_CANONICAL_WNT_SIGNALING_PATHWAY**	**1.50E-04**
POSITIVE_REGULATION_OF_VIRAL_TRANSCRIPTION	1.50E-04
CELLULAR_PROTEIN_COMPLEX_DISASSEMBLY	1.65E-04
GENE_SILENCING	1.80E-04
RRNA_METABOLIC_PROCESS	1.94E-04
PROTEASOME_ASSEMBLY	1.95E-04
TRNA_PROCESSING	2.12E-04
MACROMOLECULAR_COMPLEX_DISASSEMBLY	2.28E-04
REGULATION_OF_GENE_EXPRESSION_EPIGENETIC	3.28E-04

Online Resource 4: full list of enriched GO terms for genes more up-regulated by FGF2 (“S3” in Figure 3B)

REGULATION_OF_RNA_STABILITY	3.69E-04
PROTEIN_POLYUBIQUITINATION	3.78E-04

pathways potentially contributing to the inhibition of myelination (**bold**)
