# Supplementary material for: Polarizing receptor activation dissociates fibroblast growth factor 2 mediated inhibition of myelination from its neuroprotective potential
Source: Acta Neuropathol Commun. 2019 Dec 19;7:212. doi: 10.1186/s40478-019-0864-6 (PMC6923900; doi:10.1186/s40478-019-0864-6)
Supplement: Supplementary file 5 — Additional file 5. Online Resource 5: Pathways of genes differentially regulated in FGF2 vs F2 V2. [file 40478_2019_864_MOESM5_ESM.pdf]

Online Resource 5: pathways of genes differentially regulated in FGF2 vs F2V2

rank	pathway name	p-value	name of genes enriched
1	Rheumatoid arthritis	3.04e-06	Atp6v0d2+, Ccl5-, Ccl12+, Ccl20+, Csf1-, Cxcl12-, Flt1+, Il1a+, Il1b+, Il11+, Jun+, Mmp3+, Tcirg1-, Tgfb1+, Tgfb3+, Tnfrsf11a-, Tnfsf11+, Vegfa+
2	Cytokine-cytokine receptor interaction	4.61e-06	Bmp7+, Ccl5-, Ccl11-, Ccl12+, Ccl20+, Clcf1+, Csf1-, Cxcl11+, Cxcl12-, Cxcl13+, Cxcl16+, Cxcr4-, Egfr-, Figf+, Flt1+, Hgf-, Il1a+, Il1b+, Il1r1+, Il5+, Il11+, Lif+, Lifr-, Relt+, Tgfb1+, Tgfb3+, Tnfrsf9+, Tnfrsf11a-, Tnfrsf12a+, Tnfsf11+, Tslp+
3	Bile secretion	2.62e-05	Abcb1b+, Abcb4-, Abcc4-, Abcg2+, Adcy7+, Aqp4-, Aqp9-, Atp1a2-, Ca2+, Ephx1-, Kcnn2-, Ldlr+, Sctr+, Slc4a4-, Slc22a8-
4	Pathways in cancer	5.63e-04	Birc3+, Bmp2+, Ccne1+, Ccne2+, Cebpa+, Egfr-, Epas1-, Fgf9+, Fgf10-, Fgfr3-, Figf+, Fzd1-, Fzd10-ps1-, Hgf-, Hhip-, Itga2+, Jun+, Lef1+, Mmp2-, Mmp9+, Myc+, Nfkbia+, Prkcg-, Ptgs2+, Rad51+, Rara+, Smad3+, Tgfb1+, Tgfb3+, Vegfa+, Wnt2+, Wnt4-, Wnt7a-, Wnt7b+, Zbtb16-
5	Neuroactive ligand-receptor interaction	5.64e-04	Adra1d+, Agtr1a+, Agtr2-, Bdkrb2+, Cckbr+, Chrna3-, Crhr1+, Drd5+, F2rl1+, F2rl2+, Gabra4-, Galr1+, Gipr+, Gpr50+, Gpr83+, Grin3a-, Grm5-, Grpr+, Hcrtr1+, Hrh1+, Htr2a+, Lpar4-, Mchr1+, Nmur2-, P2rx6-, Prlhr-, S1pr1-, S1pr5-, Sctr+, Sstr4+, Tacr3+, Tshr-
6	Hepatitis C	1.18e-03	Cldn9-, Cldn11-, Cldn15+, Cldn23+, Ddx58-, Egfr-, Ifit1-, Irf1-, Irf7-, Irf9-, Ldlr+, Nfkbia+, Oas1b-, Oas2-, Ocln-, Pdk1+, Stat2-
7	Complement and coagulation cascades	1.43e-03	Bdkrb2+, C1r-, C1s-, C3-, C7-, Cd55+, F3+, Plau+, Plaur+, Pros1-, Serping1-, Thbd+
8	Malaria	1.93e-03	Ccl12+, Hgf-, Il1b+, Sdc1+, Sele+, Tgfb1+, Tgfb3+, Thbs4+, Vcam1-
9	Leukocyte transendothelial migration	1.94e-03	Cldn9-, Cldn11-, Cldn15+, Cldn23+, Cxcl12-, Cxcr4-, Ezr+, Mmp2-, Mmp9+, Myl9-, Ncf1-, Ocln-, Prkcg-, Sipa1+, Vasp+, Vcam1-
10	Glycine, serine and threonine metabolism	2.78e-03	Cbs-, Chdh-, Cth+, Gamt-, Pipox-, Sardh+, Shmt1+
11	One carbon pool by folate	2.89e-03	Dhfr+, Mthfd1l+, Mthfd2+, Mthfd2l+, Shmt1+
12	Influenza A	3.72e-03	Ccl5-, Ccl12+, Ddx58-, Hspa2-, Il1a+, Il1b+, Irf7-, Irf9-, Jun+, Map2k3+, Mx2-, Nfkbia+, Nxt1+, Oas1b-, Oas2-, Rsad2-, Stat2-, Tlr7-, Tnfrsf10b+
13	Hepatitis B	4.52e-03	Casp12-, Ccne1+, Ccne2+, Creb3l1+, Ddx58-, Egr2+, Egr3+, Irf7-, Jun+, Mmp9+, Myc+, Nfkbia+, Prkcg-, Stat2-, Stat4-, Tgfb1+, Tgfb3+
14	Nicotinate and nicotinamide metabolism	4.78e-03	Aox1-, Aox3-, Enpp1+, Nampt+, Naprt1-, Nt5c1a-
15	NF-kappa B signaling pathway	5.76e-03	Birc3+, Cxcl12-, Ddx58-, Il1b+, Il1r1+, Lyn-, Nfkbia+, Plau+, Ptgs2+, Tnfrsf11a-, Tnfsf11+, Vcam1-
16	Prion diseases	5.84e-03	C7-, Casp12-, Ccl5-, Egr1+, Il1a+, Il1b+, Notch1-
17	Basal cell carcinoma	6.27e-03	Bmp2+, Fzd1-, Fzd10-ps1-, Hhip-, Lef1+, Wnt2+, Wnt4-, Wnt7a-, Wnt7b+
18	Cytosolic DNA-sensing pathway	8.30e-03	Ccl5-, Ddx58-, Il1b+, Irf7-, Nfkbia+, Polr3g+, Ripk3+, Zbp1-
19	Focal adhesion	8.73e-03	Birc3+, Cav2-, Egfr-, Figf+, Flt1+, Hgf-, Itga2+, Itga5+, Itga7+, Itga9-, Itgb4-, Itgb5-, Jun+, Myl9-, Pdgfd-, Prkcg-, Shc4+, Thbs4+, Vasp+, Vegfa+, Zyx+
20	Bladder cancer	9.43e-03	Egfr-, Fgfr3-, Figf+, Mmp2-, Mmp9+, Myc+, Vegfa+
21	Measles	9.54e-03	Ccne1+, Ccne2+, Ddx58-, Hspa2-, Il1a+, Il1b+, Irf7-, Irf9-, Mx2-, Nfkbia+, Oas1b-, Oas2-, Stat2-, Tlr7-, Tnfrsf10b+
22	MAPK signaling pathway	1.08e-02	Bdnf+, Dusp1+, Dusp2+, Dusp4+, Dusp5+, Dusp6+, Dusp14+, Egfr-, Fgf9+, Fgf10-, Fgfr3-, Gadd45g+, Hspa2-, Hspb1-, Il1a+, Il1b+, Il1r1+, Jun+, Map2k3+, Map3k6+, Myc+, Ngf+, Prkcg-, Rasgrf2+, Tgfb1+, Tgfb3+
23	TGF-beta signaling pathway	1.15e-02	Bmp2+, Bmp6-, Bmp7+, Fst+, Inhba+, Myc+, Smad3+, Smad9-, Tgfb1+, Tgfb3+, Thbs4+
24	Arachidonic acid metabolism	1.27e-02	Cbr1+, Cbr3+, Cyp4f1-, Cyp4f4-, Cyp4f17-, Gpx2+, Pla2g5-, Ptges+, Ptgs1+, Ptgs2+, Tbxas1+
25	Arrhythmogenic right ventricular cardiomyopathy (ARVC)	1.29e-02	Dsc2+, Gja1-, Itga2+, Itga5+, Itga7+, Itga9-, Itgb4-, Itgb5-, Lef1+, Sgcg-
26	Glycerolipid metabolism	1.55e-02	Agpat2+, Akr1b8+, Akr1b10-, Gpam-, Lipg+, Mboat1+, Mgll-, Pnlip-
27	ErbB signaling pathway	1.96e-02	Areg+, Egfr-, Eif4ebp1+, Ereg+, Hbegf+, Jun+, Myc+, Nck2+, Nrg1+, Prkcg-, Shc4+
28	GnRH signaling pathway	1.96e-02	Adcy7+, Egfr-, Hbegf+, Itpr3+, Jun+, Map2k3+, Mmp2-, Mmp14+, Pld1-, Pld2-, Prkcd+
29	Dilated cardiomyopathy	1.96e-02	Adcy7+, Itga2+, Itga5+, Itga7+, Itga9-, Itgb4-, Itgb5-, Sgcg-, Tgfb1+, Tgfb3+, Tpm2+
30	Gap junction	2.13e-02	Adcy7+, Egfr-, Gja1-, Grm5-, Htr2a+, Itpr3+, Pdgfd-, Prkcg-, Tuba1c+, Tuba8+, Tubb6+
31	Leishmaniasis	2.34e-02	C3-, Il1a+, Il1b+, Jun+, Ncf1-, Nfkbia+, Ptgs2+, Tgfb1+, Tgfb3+

Online Resource 5: pathways of genes differentially regulated in FGF2 vs F2V2

**32**	Osteoclast differentiation	2.63e-02	Csf1-, Fosl1+, Il1a+, Il1b+, Il1r1+, Irf9-, Jun+, Ncf1-, Nfkbia+, Stat2-, Tgfb1+, Tnfrsf11a-, Tnfsf11+
**33**	Hematopoietic cell lineage	2.66e-02	Cd55+, Csf1-, Il1a+, Il1b+, Il1r1+, Il5+, Il11+, Itga2+, Itga5+, Mme-
**34**	Chemokine signaling pathway	2.74e-02	Adcy7+, Ccl5-, Ccl11-, Ccl12+, Ccl20+, Cxcl11+, Cxcl12-, Cxcl13+, Cxcl16+, Cxcr4-, Fgr+, Gng11+, Lyn-, Ncf1-, Nfkbia+, Prkcd+, Shc4+, Stat2-
**35**	Hypertrophic cardiomyopathy (HCM)	3.12e-02	Itga2+, Itga5+, Itga7+, Itga9-, Itgb4-, Itgb5-, Sgcg-, Tgfb1+, Tgfb3+, Tpm2+
**36**	Notch signaling pathway	3.28e-02	Dll1-, Dtx3l-, Dtx4-, Hes5-, Jag1+, Notch1-, Notch4+
**37**	Melanogenesis	3.60e-02	Adcy7+, Creb3l1+, Edn1-, Fzd1-, Fzd10-ps1-, Lef1+, Prkcg-, Wnt2+, Wnt4-, Wnt7a-, Wnt7b+
**38**	Pertussis	3.62e-02	C1r-, C1s-, C3-, Il1a+, Il1b+, Irf1-, Itga5+, Jun+, Serping1-
**39**	Chemical carcinogenesis	3.92e-02	Adh1-, Aldh1a3+, Cbr1+, Cyp3a9+, Ephx1-, Gsta3-, Hsd11b1+, Mgst1-, Ptgs2+, Sult1a1-
**40**	Transcriptional misregulation in cancer	3.96e-02	Birc3+, Cebpa+, Dusp6+, Erg+, Eya1-, Flt1+, Hmga2+, Hoxb9+, Igfbp3+, Mmp3+, Mmp9+, Myc+, Plau+, Rara+, Runx2+, Zbtb16-
**41**	Cell cycle	4.35e-02	Ccne1+, Ccne2+, Cdc6+, Gadd45g+, Mcm3+, Mcm5+, Mcm6+, Myc+, Orc1+, Smad3+, Tgfb1+, Tgfb3+, Wee1+
**42**	African trypanosomiasis	4.42e-02	F2rl1+, Il1b+, Prkcg-, Sele+, Vcam1-
**43**	Metabolism of xenobiotics by cytochrome P450	4.52e-02	Adh1-, Aldh1a3+, Cbr1+, Cbr3+, Cyp2d4-, Cyp3a9+, Ephx1-, Gsta3-, Hsd11b1+, Mgst1-
**44**	**Wnt signaling pathway**	**4.88e-02**	**Fosl1+, Fzd1-, Fzd10-ps1-, Jun+, Lef1+, Lrp5+, Myc+, Peg12+, Prkcg-, Sfrp2-, Smad3+, Wnt2+, Wnt4-, Wnt7a-, Wnt7b+**

+ up-regulated genes, - down-regulated genes
