# Supplementary material for: Polarizing receptor activation dissociates fibroblast growth factor 2 mediated inhibition of myelination from its neuroprotective potential
Source: Acta Neuropathol Commun. 2019 Dec 19;7:212. doi: 10.1186/s40478-019-0864-6 (PMC6923900; doi:10.1186/s40478-019-0864-6)
Supplement: Supplementary file 6 — Additional file 6. Online Resource 6: qPCR validation for Wnt pathway genes. [file 40478_2019_864_MOESM6_ESM.pdf]

Online Resource 6: qPCR validation for Wnt pathway genes

gene	**qPCR validation** fold change (p-value FGF2 vs F2V2)		**Microarray** fold change		**primer sequence**
	**FGF2**	**F2V2**	**FGF2**	**F2V2**	
Wnt2	1.7±0.8	0.1±0.6 (*)	2.6	-1.4	CATCTCAACAGAACCCCCTTT<br>CAGAATGCCCAAGACAGACA
Wnt7b	2.4±0.4	0.7±0.5 (*)	3.3	1.6	ACAATGAGGCAGGCAGAAAG<br>AACTTGGGCAGGGTGGTC
Fosl1	19.5±2.4	9.2±2.0 (***)	18.2	7.1	GCTGCTAAGTGCAGAAACCG<br>TGCAGCTCTTCGATCTCACG
Smad3	2.9±0.3	2.1±0.3 (*)	2.6	1.6	CTTGGCTGCTGTTTCTGTTG<br>CACTTTTGTCCCCTCTGGAA

Shown are mean +/- SEM of 7 independent experiments; p-values for fold changes of FGF2 vs F2V2 (paired t-test)
